# Acute dapsone poisoning with methemoglobinemia: a case report

**DOI:** 10.11604/pamj.2022.43.20.34069

**Published:** 2022-09-14

**Authors:** Hamza El Hamzaoui, Idriss Chajai, Mohamed Chahidi El Ouazzani, Abdelkader Benhalima, Manal El Arfaoui, Mustapha Alilou

**Affiliations:** 1 Emergency Department, Faculty of Medicine and Pharmacy of Rabat, University Mohamed V of Rabat, CHU Ibn Sina, Rabat, Morocco

**Keywords:** Poisoning, dapsone, methemoglobinemia, case report

## Abstract

Methemoglobinemia is a common complication of dapsone poisoning. Its´ treatment usually relies on methylene blue infusion. The aim of this study was to report a case of an acute dapsone poisoning with methemoglobinemia treated only with ascorbic acid and activated charcoal. A 16-year-old female voluntary ingested 3 grams of dapsone in an attempt of suicide and presented with desaturation and tachypnea. Lab findings were compatible with methemoglobinemia. After two days of treatment with ascorbic acid and activated charcoal, we observed the disappearance of desaturation and tachypnea. Methemoglobinemia can be treated with ascorbic acid and activated charcoal in limited resource settings.

## Introduction

Methemoglobinemia is defined by a level of methemoglobin superior to 1.5 g/dl [1]. It is a life threatening situation in which the severity of symptoms depends on the methemoglobin levels. It can be caused by exposure to dapsone [2]. The diagnosis relies on arterial or venous gas with co-oximetry and its management implies methylene blue infusion. We present a case of a 16-year-old female with acute dapsone poisoning presenting with methemoglobinemia, treated by ascorbic acid and activated charcoal rather than methylene blue, with a good outcome.

## Patient and observation

**Patient information:** a 16-year-old female with no medical history was admitted to the intensive care unit of the emergency department, 9 hours after the voluntary ingestion of 3 grams of dapsone, in an attempt of suicide. The parents report a violent conflict with their daughter and the patient declares that she no longer wants to commit suicide.

**Figure 1:**
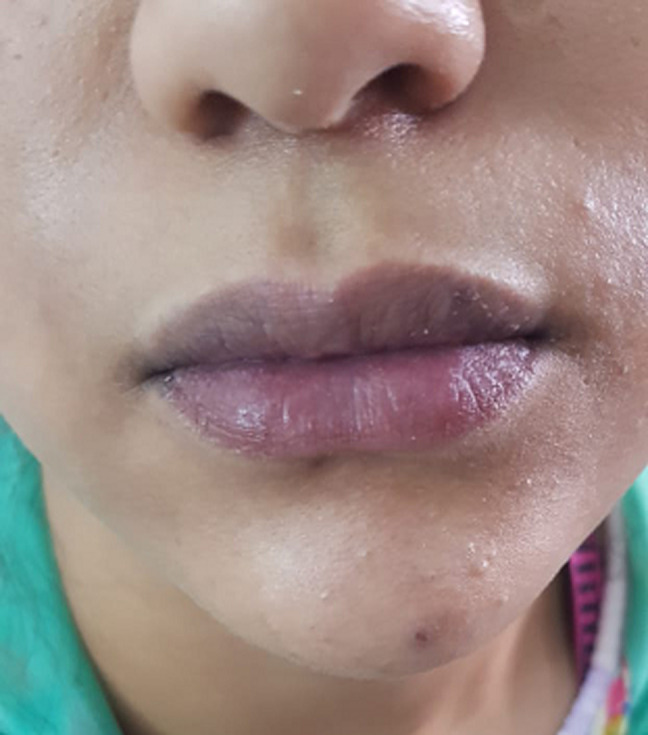
cyanosis on our patient´s lips

**Clinical findings:** the patient presented with tachypnea at 41 cycles per minute, and desaturation at 68% on room air with cyanosis (Figure 1). Her heart rate was 110 beats per minute, with a blood pressure of 114/69 with no signs of shock or cardiac failure. Pulmonary and cardiac auscultation was unremarkable. The patient was conscient and reported dizziness and headaches.

**Timeline of current episode:** 10^th^ of November 2021 at 1 a.m.: ingestion of 3 grams of dapsone; 10^th^ of November 2021 at 10 a.m.: admission of the patient with cyanosis desaturation and tachypnea, initiation of activated charcoal; 10^th^ of November 2021 at 4 p.m.: results of methemoglobin levels received, diagnosis of methemoglobinemia made; 10^th^ of November 2021 at 5 p.m.: initiation of ascorbic acid; 12^th^ of November 2021 at 11 a.m.: normal saturation, disappearance of cyanosis and tachypnea.

**Diagnostic assessment:** chest X-ray and electrocardiogram were performed with no abnormalities found. Electrolytes, renal function and the hemogram were normal (Table 1). Arterial blood gas was realized while the patient received oxygen through a non-rebreather mask and showed hypocapnia resulting from hyperventilation, with hyperoxia. The methemoglobin assay wasn´t available in our hospital´s lab, and was carried out in another laboratory. It showed a methemoglobin percentage of 13.7%.

**Table 1:** biological findings

Blood test	Results	Blood test	Results	Blood test	Results	Blood test	Results
Na^+^	139 mmol/l	pH	7.61	Hb	12.3 g/dL	Methemoglobin	13.7%
K^+^	3.7 mmol/l	PO_2_	582 mmHg	WBC	7 000/mm^3^		
Glycemia	0.89 g/dL	PCO_2_	16 mmHg	PLT	213 000/mm^3^		
Urea	0.52 g/L	BE	-2.6 mmol/L				
Creatinine	7 mg/L	HCO_3-_	15.2 mmol/L				
CK	125 UI/L	SO_2_	100%				
LDH	247 U/L						

Hb: haemoglobin; WBC: white blood cell; PLT: platelets; BE: base excess; CK: creatine kinase; LDH: lactic acid dehydrogenase

**Diagnosis:** the results were consistent with methemoglobinemia given the methemoglobin percentage of 13.7%, the saturation gap and the cyanosis.

**Therapeutic interventions:** our patient received oxygen through a non-rebreather mask. Intravenous methylene blue wasn´t available. Our patient received 1 gram of ascorbic acid twice a day orally, and a loading dose of 50 grams of activated charcoal orally, followed by 25 grams every 6 hours orally.

**Follow-up and outcome of interventions:** we observed the disappearance of cyanosis and tachypnea, with normal saturation on room air after two days of treatment after progressive weaning from oxygen. No hemolytic anemia was observed during her stay in the intensive care unit. After stabilization, the patient was transferred to the mental health unit for further management of her suicide attempt.

**Patient perspective:** “*I hope there will be no complications. Overall, the treatment wasn´t painful given it consisted mainly of oral intake*”.

**Informed consent:** the patient and her parents gave informed consent.

## Discussion

Methemoglobinemia is known to be an adverse effect of dapsone [1-3]. Its signs and symptoms are summarized in Table 2. They include headaches, cyanosis, dizziness, tachypnea and low pulse oximeter readings [3]. An important gap between SpO2 and SaO2, known as saturation gap and refractory hypoxemia measured with SpO_2_ are additional clues supporting the diagnosis of methemoglobinemia [4]. The diagnosis is confirmed by arterial or venous blood gas with co-oximetry, which determines the concentration or the percentage of methemoglobin.

**Table 2:** signs and symptoms of methemoglobinemia correlated with methemoglobin percentage in plasma

MetHb levels	Signs and symptoms
<10%	Low pulse oximeter readings, alteration of the skin color (pale gray blue)
10% - 30%	Cyanosis, dark brown blood, confusion
30% - 50%	Dyspnea, dizziness, syncope, confusion, chest pain, palpitations, headache, fatigue
50% - 70%	Tachypnea, metabolic acidosis, dysrhythmias, seizure, delirium, coma
>70%	Severe hypoxemia, death

Management of methemoglobinemia relies on methylene blue infusion. Methylene blue is an exogenous electron donor which enhances the reduction of methemoglobinemia by the action of nicotinamide adenine dinucleotide phosphate hydrogen methemoglobin (NADPH-MetHb) [4]. This treatment is contraindicated in the case of G6PD deficiency [5]. Ascorbic acid administration is considered as an alternative to methylene blue therapy, when the latter is contraindicated or not available [5]. Furthermore, dapsone is known to be adsorbed onto activated charcoal. In dapsone poisoning, multiple dose activated charcoal can be administered. It interrupts enteroenteric and hepatoenteric circulation, thus allowing secondary elimination of toxic agents [6].

In this case, the dapsone exposure, the patient´s physical examination, the saturation gap and the increased methemoglobin percentage confirmed the diagnosis of methemoglobinemia. We were limited in our approach to this case, given the unavailability of the methemoglobinemia level assay in our hospital´s laboratory and the methylene blue therapy. We initiated as fast as possible our treatment with the ascorbic acid and multiple dose activated charcoal treatment, thus improving our patient´s outcome and prognosis.

## Conclusion

Our patient with methemoglobinemia secondary to dapsone poisoning, had an excellent clinical outcome with disappearance of symptoms and signs of methemoglobinemia with the sole use of ascorbic acid and activated charcoal. This therapy can be used in the absence of methylene blue infusion or in the case of its contraindication.
